# Time in blood glucose range 70 to 140 mg/dl >80% is strongly associated with increased survival in non-diabetic critically ill adults

**DOI:** 10.1186/s13054-015-0908-7

**Published:** 2015-04-20

**Authors:** James S Krinsley, Jean-Charles Preiser

**Affiliations:** Division of Critical Care, Department of Medicine, Stamford Hospital, Columbia University College of Physicians and Surgeons, 190 West Broad Street, Stamford, CT 06902 USA; Division of Critical Care, Erasme University Hospital, Université Libre de Bruxelles, Route de Lennik 808, 1070 Brussels, Belgium

## Abstract

**Introduction:**

Hyperglycemia, hypoglycemia and increased glucose variability are independently associated with increased risk of death in critically ill adults. The relationship between time in targeted blood glucose range (TIR) and mortality is not well described and may be a factor that has confounded the results of the major interventional trials of intensive insulin therapy.

**Methods:**

We conducted a retrospective analysis of prospectively collected data involving 3,297 patients with intensive care unit (ICU) lengths of stay (LOS) of ≥1.0 day who were admitted between 1 January 2009 and 31 December 2013 to a single mixed medical-surgical ICU. We investigated the relationship between TIR 70 to 140 mg/dl with mortality and compared outcomes of non-diabetics (NON) and individuals with diabetes mellitus (DM), including stratifying by TIR above (TIR-hi) and below (TIR-lo) the median value for the NON and DM groups.

**Results:**

There were 85,799 blood glucose (BG) values for the NON group and 32,651 for the DM group, and we found that 75.5% and 54.8%, respectively, were between 70 and 140 (*P* <0.0001). The median (interquartile range) TIR (%) values for the NON and DM groups were 80.6% (61.4% to 94.0%) and 55.0% (35.5% to 71.1%), respectively (*P* <0.0001). For the NON group, mortality was 8.47% and 15.71% for TIR-hi and TIR-lo, respectively (*P* <0.0001). For the DM group, mortality was 16.09% and 14.44% for TIR-hi and TIR-lo, respectively (*P* = NS). We observed similar relationships for the NON group when we stratified by ICU LOS or severity of illness, especially in the most severely ill patients. There was a cumulative interaction of indices of hypoglycemia, hyperglycemia or glucose variability with TIR. Multivariable analysis demonstrated, for the NON group, that TIR-hi was independently associated with increased survival (*P* =0.0019). For the NON group, the observed-to-expected mortality ratios for TIR-hi and TIR-lo, based on Acute Physiology and Chronic Health Evaluation IV methodology, were 0.53 and 0.78, respectively. In contrast, among those in the DM group, there was no clear relationship between TIR 70 to 140 mg/dl and survival.

**Conclusions:**

Independently of ICU LOS and severity of illness, TIR 70 to 140 mg/dl >80% is strongly associated with survival in critically ill patients without diabetes. These findings have implications for the design of clinical protocols for glycemic control in critically ill patients as well for the design of future interventional trials of intensive insulin therapy.

**Electronic supplementary material:**

The online version of this article (doi:10.1186/s13054-015-0908-7) contains supplementary material, which is available to authorized users.

## Introduction

Time in targeted blood glucose range (TIR) may be a suitable descriptor of the efficacy and safety of glycemic control and could be considered as a marker of the severity of dysglycemia and an index of the quality of care. Neither the study that ushered in the era of “tight glycemic control” nearly 14 years ago [1] nor the trial that dampened enthusiasm for intensive control of blood glucose (BG) values 8 years later [2] reported TIR.

The Glucontrol study was the only adult randomized controlled trial (RCT) of intensive insulin therapy (IIT) that reported TIR [3]. Only 27.8% of the values obtained in patients in the experimental arm were within the targeted BG range of 80 to 110 mg/dl. Subsequent analysis of these data demonstrated that, for patients in either the intensively treated or moderate arm, with a BG target of 140 to 180 mg/dl, TIR >50% was independently associated with an increased rate of survival [4]. Chase and coinvestigators have published a series of studies that assessed the association of TIR (referred to as *cumulative time in band* with organ failure mortality in a 784-patient, before-and-after, single-center cohort evaluation of their Specialized Relative Insulin Nutrition Tables (SPRINT) protocol for IIT [5-7]. They concluded that TIR ≥50% was independently associated with less organ failure, as quantified by reduction in Sequential Organ Failure Assessment (SOFA) score [6], and that TIR ≥70% was independently associated with increased survival compared with lower thresholds of TIR (≥30% and ≥ 50%) [7]. Recently, Okabayashi and colleagues published a single-center RCT demonstrating reduction in surgical site infection with intensive vs. moderate BG targets, notable for the very high TIR achieved in the two groups with use of a closed-loop BG monitoring and insulin treatment system [8]. In contrast, another recent multicenter study in which computerized glucose control was used failed to show any clinical benefit when TIR was low [9]. These data raise the possibility that low TIR may have confounded the results of the major RCT of IIT and may explain their uneven outcomes [1-3,10,11].

A robust literature has demonstrated that hyperglycemia, hypoglycemia and increased glucose variability are independently associated with mortality in diverse cohorts of critically ill patients [12-22] and that diabetic status modulates these relationships [23-25]. The outcome of patients with diabetes mellitus (DM) may be less influenced by dysglycemia than it is among patients without diabetes (non-diabetic (NON)). Nevertheless, there is no firm consensus about how to manage glycemia in the critically ill, and some current guidelines have promoted BG targets in the hyperglycemic range to mitigate the occurrence of hypoglycemia [26-28].

We hypothesize that a high TIR is the key element of glycemic control needed to effect optimal outcome and may, in fact, blunt the deleterious impact of transient excursions into the hypoglycemic and hyperglycemic ranges. Accordingly, we evaluated the impact of a very high TIR, such as >80%, a level not generally evaluated in previous investigations, in a diverse population of critically ill patients. In addition, we assessed the interrelationship of TIR and diabetic status. To test these hypotheses, we performed a retrospective analysis of a large cohort of patients at a single mixed medical-surgical intensive care unit (ICU).

## Material and methods

### Patients and setting

The study cohort included 3,247 patients admitted to the Stamford Hospital ICU between 1 January 2009 and 31 December 2013 with an ICU LOS ≥1.0 day. During this period, there were 4,976 admissions; patients who required readmission or who had ICU LOS <1.0 day were excluded from the analysis. The ICU treats adults with a wide variety of medical and surgical conditions, including those undergoing cardiovascular surgery. The ICU has 16 beds and is staffed by full time respiratory therapists. The nurse-to-patient ratio is 1:2 or 1:1, depending on patient acuity. Stamford Hospital is a major teaching hospital affiliate of Columbia University College of Physicians and Surgeons located in Stamford, CT, USA. All orders in the ICU are written by medical or surgical residents supervised closely by medical and surgical intensivists. We abstracted information from the unit’s database. Gathered information included, in part, demographics, comorbidities, severity of illness scores, ICU LOS, ventilation metrics and hospital discharge status. Of the 3,247 patients in the study cohort, 2,550 were non-diabetic (the NON group) and 747 were diabetic (the DM group). Diabetic status was determined prospectively by the Director of Critical Care (JSK) on the basis of all clinical information available at the time of ICU admission for the vast majority of patients; in a small number of patients, diabetic status was determined retrospectively by review of the electronic medical records. The database does not include information about whether DM was categorized as type 1 or type 2.

### Glucose control

The BG target range during the study period was 90 to 120 mg/dl for all patients admitted to the ICU, a modest upward revision of the target range shown to improve mortality and morbidity of populations of critically ill patients in previously published interventional trials [5,6] (Additional file 1). This target was chosen explicitly to maximize the percentage of values within a broader 70 to 140 mg/dl range, a range that the ICU nurses felt that they could achieve. We chose this range because (1) <70 mg/dl is a widely used definition of hypoglycemia and (2) ≥140 mg/dl is a widely accepted threshold for hyperglycemia. Nurses performed BG monitoring using ACCU-CHEK Inform II glucose meters (Roche Diagnostics, Indianapolis, IN, USA) to test capillary, venous or arterial blood. Monitoring guidelines precluded use of capillary blood in the setting of shock or marked peripheral edema. The measurement frequency was every 3 hours at a minimum for all patients. Sustained hyperglycemia—two consecutive BG readings ≥180 mg/dl—triggered the institution of continuous intravenous regular insulin infusion and hourly BG measurement. The nurses treated lesser degrees of hyperglycemia with subcutaneous insulin aspart at an interval of every 3 hours. It is the standard of care in the ICU to initiate nutritional support in the first 24 to 48 hours of admission. Patients requiring more than 10 U/day of insulin who were receiving a continuous source of calories were typically administered insulin glargine to supply a portion of their daily insulin requirement. The typical starting dose of insulin glargine was one-third to one-half of the previous 24-hour insulin requirement.

### Metrics and statistical methods

We displayed continuous data as median (interquartile range (IQR)) or mean (standard deviation) and made comparisons between groups using the Mann–Whitney rank-sum test or Student’s *t*-test, respectively, as appropriate. We reported categorical data as percentages and made comparisons between groups using the χ^2^ test.

We calculated TIR 70 to 140 mg/dl as the primary outcome predictor. We calculated TIR using only recorded values, without any data extrapolation. We stratified outcomes using the median value of TIR, determined separately for the NON and DM cohorts, by creating four groups: TIR-hi and TIR-lo for the NON group and TIR-hi and TIR-lo for the DM group. We analyzed their relationship to mortality, defined as status at hospital, not ICU, and discharge, and also stratified the analysis by increments of ICU LOS and the Acute Physiology Score (APS) component of the Acute Physiology and Chronic Health Evaluation (APACHE) IV severity scoring system [29]. We constructed a multivariable model to investigate the independent association of TIR with survival. We entered parameters into the model that were statistically significant in univariate analysis at a level of *P* <0.10. The final model included age, APS, mechanical ventilation and ICU LOS. Finally, we calculated the observed-to-expected mortality ratio using APACHE IV predicted mortality.

We defined statistical significance as *P* <0.05. We performed statistical analysis using MedCalc v13.3.3 software [30].

The Stamford Hospital Institutional Review Board approved this investigation. Because of its retrospective and observational nature, informed consent of the subjects was not required.

## Results

### Clinical characteristics and glycemic control metrics for patients with or without diabetes

This investigation included 124,936 BG measurements, of which 89,803 were obtained from patients without diabetes (77.1% of the entire cohort) and 35,133 were obtained from patients with diabetes (22.9% of the entire cohort). The median (IQR) BG values for NON and DM were 119 (104 to 138) and 132 (110 to 159), respectively (*P* <0.0001).

**Figure 1:**
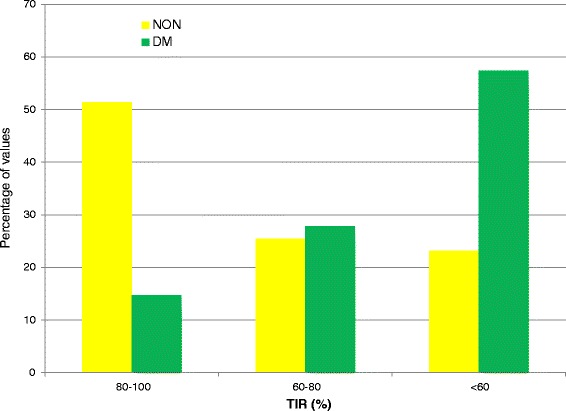
Distribution of percentages time in targeted blood glucose range for patients without or with diabetes. DM, Diabetes mellitus group; NON, Patients without diabetes mellitus; TIR, Time in targeted blood glucose range.

Figure 1 displays the TIR data. The median TIR values for the NON and DM groups were 80.6% and 55.0%, respectively. Table 1 details important clinical characteristics and glycemic control metrics of the study cohort, stratified by diabetic status. Patients with diabetes were older, had higher APS scores and APACHE IV predicted mortality, and higher mortality than did patients without diabetes. Patients with diabetes also had higher mean BG values; greater glucose variability, as reflected by higher coefficient of variation (CV); and higher rates of any and severe hypoglycemia, defined as at least one BG level <70 mg/dl or <40 mg/dl, respectively.

**Table 1:** **Clinical characteristics and glycemic control overview for patients with or without diabetes** ^**a**^

	**NON**	**DM**	***P*** **-value**
Number	2,550	747	
Age (yr)	67 (52 to 81)	70 (60 to 80)	0.0009
Median ICU LOS (IQR)	2.3 (1.5 to 5.0)	2.4 (1.6 to 5.0)	0.3004
Mean ICU LOS (SD)	4.5 (5.8)	4.6 (5.6)	N/A
Mechanical ventilation (%)	48.20	51.94	0.0790
APS	51 (37 to 70)	60 (44 to 79)	<0.0001
APACHE IV PM (%)	19.6 (23.5)^b^	25.4 (25.6)^c^	<0.0001
Mortality (%)	12.12	15.26	0.0282
TIR	80.6 (61.4 to 94.0)	55.0 (35.3 to 71.1)	<0.0001
Median number of BG tests (IQR)	17 (9 to 36)	22 (12 to 48)	<0.0001
Mean number of tests (SD)	33.6 (45.8)	43.7 (59.1)	N/A
BG tests per 24 hr	7.47	9.50	
Mean BG	121.4 (111.5 to 132.6)	140.3 (127.6 to 154.9)	<0.0001
CV (%)	17.6 (13.6 to 22.9)	27.3 (20.8 to 36.3)	<0.0001
Hypo <70 (% of patients)	18.04	31.46	<0.0001
Hypo <40 (% of patients)	1.37	2.81	0.0118

^a^Data are displayed as number, mean (SD), median (IQR) or percentage, as appropriate. Cardiovascular surgery patients did not have APACHE IV predicted mortality calculated. APACHE, Acute Physiology and Chronic Health Evaluation; APS, Acute Physiology Score component of the APACHE IV predicted mortality severity scoring system; BG, Blood glucose; CV, Coefficient of variation; DM, Diabetes mellitus group; Hypo, Hypoglycemia; ICU, Intensive care unit; IQR, Interquartile range; LOS, Length of stay; N/A, Not appropriate; NON, Non-diabetic group; PM, Predicted mortality; SD, Standard deviation; TIR, Time in targeted blood glucose range. ^b^n =2,320. ^c^n =684.

### Relationship of time in targeted blood glucose range to mortality

**Figure 2:**
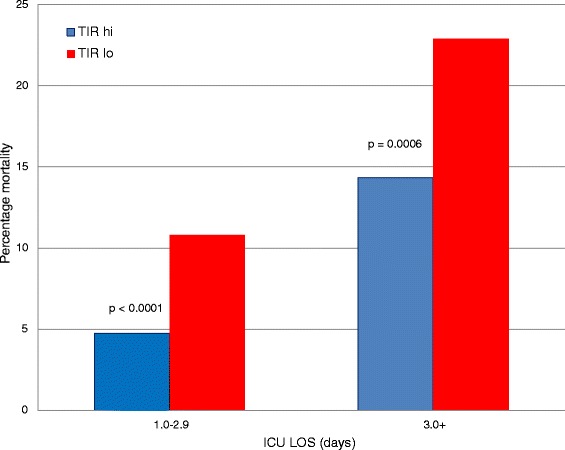
Relationship of time in range to mortality, stratified by intensive care unit length of stay, in the group of patients without diabetes. ICU LOS, Intensive care unit length of stay; TIR-hi, Time in targeted blood glucose range above median value for non-diabetic and diabetes mellitus groups; TIR-lo, Time in targeted blood glucose range below median value for non-diabetic and diabetes mellitus groups.

**Figure 3:**
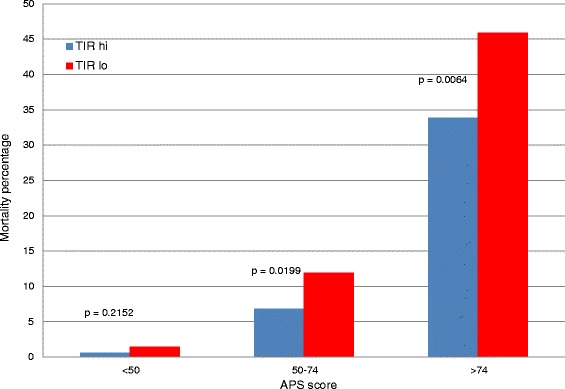
Relationship of time in range to mortality, stratified by Acute Physiology Score, in the group of patients without diabetes. APS, Acute Physiology Score component of the Acute Physiology and Chronic Health Evaluation IV severity scoring system; TIR-hi, Time in targeted blood glucose range above median value for non-diabetic and diabetes mellitus groups; TIR-lo, Time in targeted blood glucose range below median value for non-diabetic and diabetes mellitus groups.

Table 2 details important clinical characteristics and glycemic control metrics of the study cohort for TIR-hi and TIR-lo, stratified by diabetic status. In the NON group, mortality was nearly twice as high for TIR-lo as for TIR-hi, whereas the difference in mortality between TIR-hi and TIR-lo was not significant in the DM group. We constructed two sensitivity analyses. Figure 2 demonstrates that, for the NON group, TIR-lo was strongly associated with increased mortality compared with TIR-hi, especially with longer ICU LOS. Figure 3 demonstrates that TIR-lo was strongly associated with increased mortality compared with TIR-hi, especially with higher APS scores. Among the DM group, there was no significant relationship between TIR and mortality.

**Table 2:** **Comparison of time above and below targeted blood glucose range for patients with or without diabetes, as well as clinical characteristics and glycemic control metrics** ^**a**^

			**TIR-hi**	**TIR-lo**	***P*** **-value**
NON group					
	Clinical parameters				
		Age (yr)	65 (48 to 80)	70 (56 to 82)	<0.0001
		Median ICU LOS (IQR)	2.2 (1.4 to 5.0)	2.4 (1.6 to 5.0)	0.0657
		Mean ICU LOS (SD)	4.4 (5.6)	4.6 (5.9)	N/A
		Mechanical ventilation (%)	45.18	51.22	0.0026
		APS	48 (34 to 67)	54 (40 to 74)	<0.0001
		APACHE IV PM (%)	16.6 (20.9)	22.9 (25.7)	<0.0001
		Mortality (%)	8.47	15.76	<0.0001
	Glucose metrics				
		TIR (%)	94.0 (87.8 to 100.0)	61.4 (46.5 to 71.4)	<0.0001
		Median number of BG tests (IQR)	15 (9 to 35)	18 (10 to 39)	0.0001
		Mean number of tests (SD)	30.7 (40.5)	36.6 (50.4)	N/A
		BG tests per 24 hr	7.0	8.0	
		Mean BG (SD)	113.4 (105.3 to 119.4)	131.9 (123.7 to 140.7)	<0.0001
		CV (%)	15.0 (11.6 to 19.2)	20.8 (16.3 to 25.8)	<0.0001
		Hypo <70 (% of patients)	16.63	19.45	0.0718
		Hypo <40 (% of patients)	1.10	1.65	0.3066
DM group					
	Clinical parameters				
		Age (yr)	71 (61 to 80)	69 (59 to 80)	0.2418
		Median ICU LOS (IQR)	2.7 (1.6 to 7.4)	2.1 (1.4 to 3.8)	0.0001
		Mean ICU LOS (SD)	5.6 (6.5)	3.6 (4.4)	N/A
		Mechanical ventilation (%)	60.32	43.58	<0.0001
		APS	63 (46 to 83)^b^	57 (44 to 75)^c^	0.0175
		APACHE IV PM (%)	27.9 (25.4)^d^	22.7 (25.4)^e^	0.0071
		Mortality (%)	16.09	14.44	0.5994
	Glucose metrics				
		TIR (%)	71.1 (63.4 to 82.0)	35.4 (24.1 to 47.3)	<0.0001
		Median number of BG tests (IQR)	24 (13 to 62)	20 (12 to 36)	0.0023
		Mean number of tests (SD)	52.0 (68.7)	35.4 (46.3)	N/A
		BG tests per 24 hr	9.3	9.8	
		Mean BG (SD)	129.6 (119.9 to 138.8)	151.7 (142.1 to 166.7)	<0.0001
		CV (%)	25.2 (19.3 to 33.8)	29.5 (22.2 to 37.7)	<0.0001
		Hypo <70 (% of patients)	38.07	24.87	0.0001
		Hypo <40 (% of patients)	2.95	2.67	0.9919

^a^Data are displayed as number, mean (SD), median (IQR) or percentage, as appropriate. Cardiovascular surgery patients did not have APACHE IV predicted mortality calculated. APACHE, Acute Physiology and Chronic Health Evaluation; APS, Acute Physiology Score component of the APACHE IV predicted mortality severity scoring system; BG, Blood glucose; CV, Coefficient of variation; DM, Diabetes mellitus group; Hypo, Hypoglycemia; ICU, Intensive care unit; IQR, Interquartile range; LOS, Length of stay; N/A, Not appropriate; NON, Non-diabetic group; PM, Predicted mortality; SD, Standard deviation; TIR, Time in targeted blood glucose range; TIR-hi, Time in targeted blood glucose range above median value for non-diabetic and diabetes mellitus groups; TIR-lo, Time in targeted blood glucose range below median value for non-diabetic and diabetes mellitus groups. ^b^n =1,227. ^c^n =343. ^d^n =1,099. ^e^n =340.

### Interaction of domains of glycemic control

Table 3 illustrates the interaction of TIR and three domains of glycemic control in the NON and DM groups: hypoglycemia, defined as at least one BG value <70 mg/dl; hyperglycemia, defined as at least one BG value ≥180 mg/dl; and increased glucose variability, defined as a CV ≥20%. These data demonstrate a strong interaction between metrics of glycemic control in the NON group, but not in the DM group. In the NON group, even among those with high TIR, the presence of concomitant hypoglycemia, hyperglycemia or increased glucose variability was associated with increased risk of death.

**Table 3:** **Interaction of domains of glycemic control with time in targeted blood glucose range** ^**a**^

			**Number**	**Mortality (%)**	***P*** **-value**
NON group					
	TIR-hi				
		Min BG ≥70 mg/dl	1,063	6.49	<0.0001
		Min BG <70 mg/dl	212	18.40	
		CV <20%	1,007	6.85	<0.0001
		CV ≥20%	268	15.30	
		Max BG <180 mg/dl	1,041	6.82	<0.0001
		Max BG ≥180 mg/dl	234	15.81	
	TIR-lo				
		Min BG ≥70 mg/dl	1,027	11.39	<0.0001
		Min BG <70 mg/dl	248	33.87	
		CV <20%	585	9.23	<0.0001
		CV ≥20%	690	21.30	
		Max BG <180 mg/dl	474	10.76	0.0002
		Max BG ≥180 mg/dl	801	18.73	
DM group					
	TIR-hi				
		Min BG ≥70 mg/dl	231	10.82	0.0007
		Min BG <70 mg/dl	142	24.65	
		CV <20%	102	10.78	0.1206
		CV ≥20%	271	18.08	
		Max BG <180 mg/dl	94	14.89	0.8392
		Max BG ≥180 mg/dl	279	16.49	
	TIR-lo				
		Min BG ≥70 mg/dl	281	14.23	0.9802
		Min BG <70 mg/dl	93	15.05	
		CV <20%	61	14.75	0.9014
		CV ≥20%	313	14.38	
		Max BG <180 mg/dl	28	14.29	0.7977
		Max BG ≥180 mg/dl	346	14.45	

^a^BG, Blood glucose; CV, Coefficient of variation; DM, Diabetes mellitus group; Max, Maximum; Min, Minimum; NON, Non-diabetic group; TIR, Time in targeted blood glucose range 70 to 140 mg/dl; TIR-hi, Time in targeted blood glucose range above median value for non-diabetic and diabetes mellitus groups; TIR-lo, Time in targeted blood glucose range below median value for non-diabetic and diabetes mellitus groups.

### Multivariate analysis

Table 4 displays the results of multivariable analysis. Among the NON group, TIR-lo was independently associated with increased risk of death, with a 61% increase in the odds of mortality. In contrast, there was no independent association of TIR-lo with mortality among the DM group.

**Table 4:** **Multivariable analysis: association with mortality** ^**a**^

**Variable**		**Coefficient**	**Standard error**	**OR (95% CI)**	***P*** **value**
NON group					
	Age	0.0213	0.0049	1.02 (1.02 to 1.03)	<0.0001
	APS	0.0499	0.0031	1.05 (1.04 to 1.06)	<0.0001
	Mechanical ventilation	0.7025	0.1794	2.02 (1.42 to 2.87)	0.0001
	TIR-lo	0.4744	0.1531	1.61 (1.19 to 2.17)	0.0019
DM group					
	Age	0.0364	0.0102	1.04 (1.02 to 1.06)	0.0004
	APS	0.0532	0.0054	1.05 (1.04 to 1.07)	<0.0001
	Mechanical ventilation	0.6886	0.3177	1.99 (1.07 to 3.71)	0.0302
	TIR-lo	0.0780	0.2610	1.08 (0.65 to 1.80)	0.7649

^a^Age, OR per 1 year. APS, OR per 1 point. APS, Acute Physiology Score component of the Acute Physiology and Chronic Health Evaluation IV predicted mortality severity scoring system; CI, Confidence interval; DM, Diabetes mellitus group; NON, Non-diabetic group; OR, Odds ratio; TIR-hi, Time in targeted blood glucose range above median value for non-diabetic and diabetes mellitus groups; TIR-lo, Time in targeted blood glucose range below median value for non-diabetic and diabetes mellitus groups.

**Figure 4:**
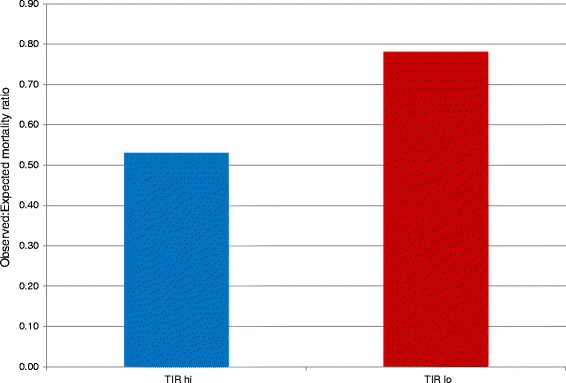
Observed-to-expected mortality ratio for of time above and below targeted blood glucose range in the group of patients without diabetes. TIR-hi, Time in targeted blood glucose range above median value for non-diabetic and diabetes mellitus groups; TIR-lo, Time in targeted blood glucose range below median value for non-diabetic and diabetes mellitus groups.

Finally, Figure 4 corroborates these findings by illustrating the substantially lower observed-to-expected mortality ratio in the NON group, using APACHE IV methodology, of TIR-hi compared with TIR-lo.

## Discussion

In this retrospective analysis of a large, heterogeneous cohort of critically ill patients, we investigated the relationship between TIR 70 to 140 mg/dl and mortality. The salient finding of the study is that TIR was strongly associated with survival among patients without diabetes, with a 61% increase in the odds of death when we compared patients below and above the median value for TIR. The association between TIR and mortality was independent of severity of illness, with the greatest difference demonstrated in the subpopulation with the highest severity of illness. In addition, the association between TIR and mortality was independent of ICU LOS. Notably, TIR-hi was strongly associated with survival among patients with ICU LOS of 1 to 3 days; earlier data derived from interventional trials suggested no benefit of glycemic control in a short-stay cohort [1,10].

In contrast, among patients with DM in the present study, there was no consistent relationship between TIR and mortality. Glycemic control differed substantially among patients with vs. without DM. Although BG targets and glycemic control guidelines in the ICU did not differ based on diabetic status, patients with DM had higher mean BG, higher glucose variability, higher rates of hypoglycemia and a much lower TIR than did those without DM. These divergent findings are consistent with recent literature describing differences between patients with vs. without diabetes regarding the relationships of hyperglycemia, hypoglycemia and glucose variability to mortality [23-25].

These data expand on the limited literature that has explored the relationship between TIR and mortality in critically ill patients. Chase and colleagues evaluated the clinical impact of a computerized glycemic control protocol requiring BG measurement every 1 to 2 hours that controlled insulin infusion and nutrition input concomitantly [5]. Using a before-and-after experimental design, they demonstrated reduced mortality in the interventional group among those staying in the ICU for 3 to 5 days [6]. Subsequent analysis included the entire cohort of 784 patients to investigate the relationship between TIR 72 to 126 mg/dl and clinical endpoints [6,7]. TIR ≥50% was independently associated with reduced organ failure, as shown by fewer patients with SOFA scores ≤5 after day 5 in the ICU [6]. In addition, TIR ≥70% was independently associated with increased odds of survival compared with TIR ≥50% or TIR ≥30% [7]. These investigations did not stratify patients by diabetic status (17% of the entire cohort).

Our findings shed light on the varied results of the interventional trials of IIT. Intensive monitoring of BG levels and treatment of hyperglycemia became a worldwide standard of care following publication of a single-center study conducted in a population of mechanically ventilated surgical patients that demonstrated marked reductions in mortality and morbidity in patients treated with intravenous insulin and a BG target of 80 to 110 mg/dl [1]. Subsequent interventional trials failed to reproduce these impressive findings [2,3,10,11]. Only one of these studies explicitly reported TIR [3]; in fact, the Data Safety Monitoring Board for that study forced the premature termination of the study because of the low TIR. Estimates of TIR in the intensive insulin arm for the major interventional trials, inferred using the reported morning BG values and the standard normal distribution table, range from 31% [2] to 53% [1]. However, the comparison of TIR of these studies is confounded by the differences in the number of BG values used and in BG monitoring, including the use of arterial blood and blood gas analyzers [1] or combinations of arterial blood, capillary blood, blood gas analyzers and bedside glucometers [2,3,10,11]. None of these studies reported BG monitoring frequency.

In contrast to these findings, Okabayashi and coworkers performed a single-center RCT in 447 patients undergoing hepatobiliary or pancreatic surgery to compare infection rates in those with BG targets of 80 to 110 mg/dl and 140 to 180 mg/dl, the same targets used in the Normoglycemia in Intensive Care Evaluation and Surviving Using Glucose Algorithm Regulation trial (referred to as NICE-SUGAR) [8]. They used a computerized, closed-loop BG monitoring and insulin delivery system to achieve very high TIR for the two study groups—85.8% and 96.8%, respectively—without significant differences between patients with and without DM. Notably, surgical site infection, the primary outcome, occurred in 9.8% of patients treated with the intermediate BG target and 4.1% of those treated with the “tight” BG target (*P* =0.028). This intriguing investigation is, in fact, hypothesis-generating. Although a high rate of hypoglycemia in the intensively treated patients of the major interventional trials of IIT has been independently associated with increased risk of death in all of the studies [1-3,10,11,14,15], and though elevated glucose variability has been independently associated with increased risk of death in two of them [14], it is quite possible that the low TIR contributed to the finding of lack of benefit of the intervention. This conclusion is supported by a recently published *post hoc* analysis of the multicenter Glucocontrol trial of IIT [4]. The Glucocontrol trial was terminated prematurely because of the low rate of achieving the targeted BG for the patients in the interventional arm. The new analysis concludes that, though there was no difference in the development of organ failure based on intention to treat to different glycemic targets (BG 80–110 mg/dl in the intensive arm and 140 to 180 mg/dl in the conventional arm), achieving the BG range of 72 to 136 mg/dl was independently associated with increased survival for patients in either arm of the trial.

The present study has several strengths. The large dataset is robust, containing a comprehensive set of clinical parameters. The patients were treated consistently over the period of time they were included in the study, with a high frequency of BG measurements exceeding that commonly seen in clinical practice [22]. The analysis includes an explicit detailing of the relationship of TIR to survival, as well as its interaction with the other domains of glycemic control: hyperglycemia, hypoglycemia and glucose variability. Finally, the investigation stratifies patients by diabetic status, a factor increasingly recognized in the literature regarding glycemic control in critically ill patients [23-25].

There are several limitations of the present study. First, the major limitation is its observational nature. Consequently, its conclusions must be considered hypothesis-generating rather than as proof of causality. Nevertheless, it is unlikely that a randomized trial will ever be conducted that stratifies patients into a TIR-lo cohort. Second, we conducted a single-center study, raising questions about its generalizability. However, the ICU treats a heterogeneous population of critically ill adults admitted with a wide array of medical and surgical diagnoses. Third, there is significant mathematical coupling between TIR and the other established domains of glycemic control hyperglycemia, hypoglycemia and glucose variability. A patient with low TIR is certainly more likely to have higher rates of dysglycemia reflected by the other three glucose metrics. Nevertheless, the data in Table 3 demonstrate, especially for non-diabetics, that concomitant hypoglycemia, hyperglycemia or increased glucose variability, regardless of TIR, was associated with increased risk of death. Fourth, we do not have hemoglobin A1c values for the patients. Some patients characterized as non-diabetic may, in fact, have more properly been characterized as previously undiagnosed diabetics. Fifth, we do not provide data about nutritional support or insulin therapy; subsequent work may help delineate the interplay between these factors and glycemic control. Finally, the ICU staff employed glucometers for BG analysis, using capillary blood in the majority of tests and venous or arterial blood in the remaining. Although this method of analysis has been associated with analytic inaccuracies [31-33], it remains the standard of care in many ICUs, especially in the United States.

These findings have implications relating to current clinical practice, to the assessment of quality of care and for the design of future interventional trials using new methods to improve glucose control. We have shown that TIR 70 to 140 mg/dl is independently associated with survival in a heterogeneous cohort of critically ill patients without diabetes. Nevertheless, one of the lessons of the interventional trials of glycemic control is that high TIR is an elusive target that is very difficult to achieve. Computer-guided insulin administration algorithms may be one way to increase TIR and consequently improve glycemic control. The Leuven investigators have demonstrated that use of a computer-guided algorithm increased TIR from 60.1% to 68.8% compared with the BG control efforts of their well-trained nurses [34]. Similarly, Juneja and coworkers used a software program to guide insulin therapy targeting 80 to 110 mg/dl in 4,488 ICU patients, achieving TIR of 73.4% [35]. Nevertheless, 4.2% of patients sustained at least one episode of severe hypoglycemia (BG <40 mg/day). The investigators concluded that measurement delay (mean of only 12 minutes, with a protocol requiring hourly monitoring) was contributory in two-thirds of these episodes.

Importantly, even hourly BG monitoring is associated with a high likelihood of missed excursions into the hyperglycemic and hypoglycemic ranges. This has been clearly demonstrated in studies in which researchers investigated continuous or near-continuous BG monitors [36,37], suggesting that increased BG monitoring frequency may provide the best path to achieving high TIR. Indeed, in a recently published Monte Carlo simulation of the impact of monitoring frequency on the occurrence of hyperglycemia and hypoglycemia, investigators determined that rates of dysglycemia decreased substantially when monitoring frequency was increased from every 2 hours to every 1 hour, with further modest improvement resulting from an increase in frequency to every 15 minutes [38].

These data suggest the possibility that TIR may be a useful quality-of-care metric for assessing glucose control practice in an ICU in view of its ease of measurement and strong association with risk of death. Finally, we believe that the findings of the present study have important implications for the design of future interventional trials of IIT in critically ill patients. First, our data confirm that the optimal target for BG control in critically ill patients with diabetes has not yet been established, a finding that is consistent with recent observational literature [23-25]. Clinical equipoise may now exist for the implementation of an interventional trial incorporating multiple BG targets, based on patient characteristics, including diabetic status and, for patients with diabetes, preexisting glycemic control [39,40] Most importantly, achieving a high TIR, such as >80%, must be considered as integral to the success of the trial. Notably, the number of patients needed for a trial will likely be much lower than the numbers participating in the previously published interventional trials if TIR is very high, as demonstrated by a recently published study using a closed-loop insulin delivery system that in which researchers found a reduction in surgical site infection when they compared intensive and moderate BG targets, with just over 200 patients in each arm of the study [8]. These findings cannot be achieved with the monitoring technologies and the monitoring frequencies used in the published interventional trials of IIT [41]. In future trials, investigators must consider use of the new emerging technologies that allow continuous or near-continuous BG monitoring.

## Conclusions

In this retrospective evaluation of a heterogeneous cohort of critically ill patients, we demonstrate that TIR 70 to 140 mg/dl was independently associated with survival among patients without diabetes. There was a 61% increase in the odds of death among non-diabetics when we compared patients below and above the median value for TIR. This association was seen consistently, regardless of ICU LOS, including those with short ICU LOS, and was strongest for those patients with the highest severity of illness. The relationship between TIR and mortality among patients with diabetes was less clear, and glucose control among this group of patients was challenging, with higher mean BG, higher glucose variability and higher rates of hypoglycemia compared with results in patients without diabetes, even though the number of BG measurements was higher among patients with diabetes than in those without diabetes. These findings have important implications for current clinical practice as well as for the design of future interventional trials of IIT in critically ill patients.

## Key messages

- TIR 70 to 140 mg/dl is independently associated with survival in critically ill patients without diabetes, with a nearly twofold increase in mortality between patients below and above the median value for TIR, 80.6%.
- This relationship is independent of ICU LOS and severity of illness.
- The metrics of glycemic control in individuals without diabetes, including TIR, hypoglycemia, hyperglycemia and glucose variability, exceed those seen in patients with diabetes.
- These findings have important implications for current clinical practice as well as for the design of future interventional trials of IIT in critically ill patients.

## Additional file

Additional file 1:
**Glucose management protocol.** The insulin treatment guideline in use in the Stamford Hospital ICU during the period of the study.

## Abbreviations

APACHE: Acute Physiology and Chronic Health Evaluation
APS: Acute Physiology Score
BG: Blood glucose
CI: Confidence interval
CV: Coefficient of variation
DM: Diabetes mellitus
Hypo: Hypoglycemia
ICU: Intensive care unit
IIT: Intensive insulin therapy
IQR: Interquartile range
LOS: Length of stay
N/A: Not appropriate
NICE-SUGAR: Normoglycemia in Intensive Care Evaluation and Surviving Using Glucose Algorithm Regulation
NON: Non-diabetic
OR: Odds ratio
RCT: Randomized controlled trial
SD: Standard deviation
SOFA: Sequential Organ Failure Assessment
SPRINT: Specialized Relative Insulin Nutrition Tables
TIR: Time in targeted blood glucose range
TIR-hi: Time in targeted blood glucose range above median value for non-diabetic and diabetes mellitus groups
TIR-lo: Time in targeted blood glucose range below median value for non-diabetic and diabetes mellitus groups
