# Supplementary material for: Time in blood glucose range 70 to 140 mg/dl >80% is strongly associated with increased survival in non-diabetic critically ill adults
Source: Crit Care. 2015 Apr 20;19(1):179. doi: 10.1186/s13054-015-0908-7 (PMC4446958; doi:10.1186/s13054-015-0908-7)
Supplement: Additional file 1: — Glucose management protocol. The insulin treatment guideline in use in the Stamford Hospital ICU during the period of the study. [file 13054_2015_908_MOESM1_ESM.doc]

| BG (mg/dL) | SC Aspart Insulin Units |
| --- | --- |
| > 300 | Insulin gtt |
| 250-299 | 10 |
| 200-249 | 8 |
| 175-199 | 6 |
| 150-174 | 4 |
| 135-149 | 2 |
| 120-134 | 1.5 |

| BG (mg/dL) | Management of BG <120 mg/dL on an Insulin Drip |
| --- | --- |
| 80-119 | Continue insulin infusion at 1 unit/hr. |
| 60-79 | Stop insulin infusion, recheck in 1 hr |
| 40-59 | Stop infusion and initiate D10 at 100ml/hr recheck BG in 30min and 60 min. Stop infusion when BG >79. |
| <40 | Stop infusion, give1/2 amp D50 give recheck in 30 min |


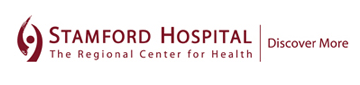


| BG (mg/dL) | Management of BG<80mg/dl on sc Insulin |
| --- | --- |
| 60-79 | If patient is asymptomatic, recheck BG in 1 hour. If symptomatic, treat patient using the 40-59 mg/dl guideline. |
| 40-59 | If patient is ordered for PO intake, give 120ml of apple juice, if NPO initiate D10 at 100ml/hr and recheck BG in 30 min and 60 min. Stop infusion when BG >79. |
| <40 | Give 1/2 amp D50, check BG in 30min. Notify MD. |

**Important Points:**

- Protocol is initiated by RN, when there is one BG > 300, or two consecutive BG > 175. MD must be notified regarding initiation of insulin drip. IV regular insulin is administered at concentration of 1unit/mL.
- All patients receiving continuous insulin with a BG <200 must receive a continuous source of glucose, either via D5W, D10W, TPN or enteral feeds.
- BG monitoring frequency is Q1 hr.
- Patients with severe shock or severe edema should have BG monitored using venous or arterial blood, rather than fingerstick capillary blood.
- If BG has not decreased after 2 BG, insulin dose should be increased.
- If BG control stabilizes (4 consecutive BG between 90-120 mg/dl) on the infusion, monitoring frequency can be decreased to Q2hours.
- Variances to the protocol are documented by the RN in Meditech and the MD is notified.

**RISK OF HYPOGLYCEMIA INCREASES:**

- In patients not receiving nutrition. Consider holding insulin in this case.
- In patients with ESRD or liver failure. Treat initially with 50% suggested insulin dose and consider increasing the frequency of BG monitoring.

**ICU and IMCU Insulin Drip Protocol**

**Goal: To bring blood glucose (BG) to a level between 90-120 mg/dl**

**When BG rate of change >50mg/dL:**

- If BG is between 200-300 with greater than 100 BG drop since the previous BG, decrease insulin dose by 50% and recheck BG in 1 hour.

- If BG is between 150-200 with greater than 50 BG drop since the previous BG decrease insulin dose by 50% and recheck BG in 1 hour.

**This protocol is not to be used for patients being treated for diabetic ketoacidosis.**

**These guidelines can be modified if the patient requires more or less intensive therapy.**

| BG (mg/dL) | Insulin Gtt Rate units/hr |
| --- | --- |
| > 400 | 12 |
| 350-399 | 10 |
| 300-349 | 9 |
| 275-299 | 8 |
| 250-274 | 7 |
| 225-249 | 6 |
| 200-224 | 5 |
| 175-199 | 4 |
| 150-174 | 3 |
| 135-149 | 2 |
| 120-134 | 1.5 |

| Transition to Subcutaneous Therapy |
| --- |
| - For patients with **low (requiring < 1 units/hr)** but stable (90-119 mg/dl) insulin requirement turn off insulin drip.  - For patients with **high (requiring > 2 units/hr)** but stable insulin requirement on Insulin drip, calculate previous 24 hour total insulin given and divide by 2 to calculate dose of Lantus. Give 50% of calculated dose at 0800 or 2000, then turn off Insulin drip 2 hours after giving lantus dose. |
| Shut off D5W once insulin drip is shut off. Check blood sugar in 2 hrs and begin subcutaneous insulin protocol. |
| The transition to subcutaneous therapy is based on the patient’s overall clinical status, not just the degree of BG control. Unstable patients (eg shock) should have the insulin infusion continued even if excellent BG control has been achieved with a low insulin infusion rate. |

**ICU and IMCU Subcutaneous Insulin Protocol**

**Goal: To bring blood glucose (BG) to a level between 90-120 mg/dl**

**Important Points:**

- Protocol is initiated upon patient’s admission to ICU, or transition from IV insulin drip. Aspart insulin is the designated sc insulin.
- BG monitoring frequency is Q3 hr unless the patient is on po diet, in which case BG is monitored AC/HS.

-Patients with severe shock or severe edema should have BG monitored using venous or arterial blood, rather than fingerstick capillary blood.

- If the daily Aspart dose exceeds 15 units, addition of Glargine insulin should be considered. Glargine should be administered Q12 hrs. The starting Glargine dose should be 33% - 50% of the previous day’s total Aspart dose.
- Oral hypoglycemic agents should not be used in the ICU.
- If patient’s BG remains in the 90-120 mg/dL range without iinsulin requirement for 48 hours, BG monitoring frequency can be decreased to Q6hrs.
- Variances to the protocol are documented by the RN in Meditech and the MD is notified.
